# Effects of a methionine deficiency on chicken tissue protein turnover: comparative analysis of methionine source

**DOI:** 10.1016/j.psj.2025.105410

**Published:** 2025-06-07

**Authors:** José Alberto Conde-Aguilera, Sonia Métayer-Coustard, Yves Mercier, Jaap van Milgen, Sophie Tesseraud

**Affiliations:** a INRAE, Université de Tours, BOA 37380 Nouzilly, France; b Adisseo France SAS, E.L.I.S.E., Department of R&I in Monogastric Animal Nutrition, Saint Fons, France; c INRAE, Agrocampus Ouest, PEGASE 35590 Saint-Gilles, France

**Keywords:** Methionine, Protein synthesis, Protein degradation, Sulphur AA, Poultry

## Abstract

Methionine (**Met**) supply is critical for optimum growth rate, muscle development, antioxidant activity, one-carbon metabolism in liver, and feather production in birds. Met is the first limiting amino acid (**AA**) in chicken diets, yet little is known about how different Met levels and sources influence tissue protein metabolism. Growth performance and tissue protein metabolism were compared in broilers fed either deficient or sufficient in Met, supplemented with either DL-Met or DL-HMTBA (a Met analogue). Protein synthesis rates were quantified *in vivo* in the liver, in 2 muscle types, and in the jejunum of 3-week-old chickens using a flooding dose of [^13^C]-Valine. Additionally, tissue proteolytic activities and the expression of genes involved in proteolysis and autophagy were measured. The deficient Met supply reduced chicken weight, *Pectoralis major* (**PM**) muscle weight and protein synthesis (*P*<0.05), though liver weight and protein synthesis remained unaffected. When expressed as fractional synthesis rates (**FSR**, %/d), protein synthesis was never depressed by Met deficiency irrespective of the tissue studied, whereas the proteasome proteolytic activity was consistently greater (*P*<0.01) in the Met-deficient birds. These results were supported by the expression of proteolysis-related genes in the PM. For most parameters, including FSR and proteolytic activities (with the exception of the liver), there were minimal differences between birds receiving DL-Met and DL-HMTBA, indicating that DL-HMTBA effectively supports muscle protein synthesis when adequately supplied, similar to DL-Met. In conclusion, Met deficiency profoundly impacts chicken growth, PM development, and tissue protein turnover, while the two Met sources exhibit similar effects.

## Introduction

Methionine (**Met**) and cysteine (**Cys**) hold very important places among amino acids (**AA**) by playing numerous roles in metabolism and cell functions (Hou et al., 2020; Kožich and Stabler, 2020; Wu, 2009). Like other AA, they are part of tissue proteins, serving as substrates for protein synthesis. A key role of Met is its involvement in one-carbon (1C) metabolism providing 1C units to various biosynthetic pathways, thereby regulating purine, thymidine and polyamines synthesis, AA homeostasis, and epigenetic mechanisms (Clare et al., 2019; Tesseraud et al., 2009; Wu, 2009). Additionally, Met is involved in the synthesis of other sulphur-containing AA, notably Cys through transsulfuration. Cys, in turn, is required for the production of essential molecules like glutathione (Cys-Glu-Gly) and taurine, which play vital roles in protecting against oxidative stress (Brosnan and Brosnan, 2006; Métayer et al., 2008; Shoveller et al., 2005).

Amongst essential AA, Met is typically the first limiting AA in broiler diets, directly influencing growth and development (Baker et al., 2006). This is especially critical in poultry due to the high Cys requirements for feather synthesis. Consequently, poultry diets are commonly supplemented with either Met or its hydroxy analogue, 2-hydroxy-4-methylthio butyric acid (**HMTBA**), to meet chicken requirements. Despite its widespread use, the relative biological efficacy of DL-HMTBA compared to DL-Met has been a topic of debate for over 50 years (Agostini et al., 2016; Baker, 2009; Becquet et al., 2023). This discrepancy is likely due to methodological variations in studies that overlook the distinct conversion and metabolism of the 3 enantiomers (L-HMTBA, D-HMTBA, and D-Met) into L-Met (see Becquet et al., 2023 for a recent review). Some studies, for example, have focused on the differences in Met absorption or its incorporation into tissues by using isotopic labelled Met, such as stable carbon (^13^C) or radioactive isotopes of hydrogen (^3^H) and carbon (^14^C). However, these approaches fall short of providing a complete picture of the bioavailability of Met sources, as they primarily reflect their absorption and metabolism rather than the overall utilization of Met by the animal.

While dietary sulphur AA have been extensively studied, there is limited understanding on how different sources and levels of supply influence tissue composition and protein metabolism. Previous studies in chickens have demonstrated that supplementing a protein-free diet with Met increases the synthesis rates (in %/d) of body proteins, particularly muscle proteins (Muramatsu et al., 1985; 1986). Although surprising considering that Met is the only AA provided in such a diet, this effect is likely due to an enhanced reutilization rate of endogenous-formed AA originating from body protein degradation, thereby improving N balance and reducing weight loss in chickens fed protein deficient diets supplemented with Met. Other studies designed to have varied Met levels in diets balanced for other AA showed increased muscle protein synthesis in both chickens (Barnes et al., 1995) and piglets (Conde-Aguilera et al., 2016b). However, the effect of Met supplementation appears to depend on muscle type. Additionally, Met provision has been shown to influence the chemical composition of tissues and certain aspects of meat quality (Conde-Aguilera et al., 2013). The source of Met can also affect body AA composition and the distribution of Cys among tissues, providing further insight (Conde-Aguilera et al., 2016a).

Building on the previous works, the current study aims to investigate the effects of different Met sources on protein metabolism in growing broilers under deficient Met supplies. We measured protein synthesis in the *Pectoralis major* (**PM**) muscle (a fully fast-twitch glycolytic muscle), thigh muscles (mixed-type muscles), jejunum and liver, using the flooding-dose method (Garlick et al., 1994; flooding dose of [^13^C]-Val here), which is considered the most reliable technique for measuring tissue protein synthesis rates *in vivo* (see critical analysis by Garlick et al., 1994). Additionally, we analysed tissue proteolytic activities, expression of genes related to proteolysis and autophagy, as well as muscle peptide and free AA composition.

## Materials and methods

### Experimental design, diets and feeding

Experimental procedures and animal care were carried out following the European legislation (EU Directive 2010/63/EU). The local Ethical Committee for Animal Experimentation of Val de Loire (**CEEA Vdl**) and the French Ministry of Research considered this experiment beyond the scope of the European and French animal experimentation regulations (Article R214–98 of the Code rural et de la pêche maritime transposing the Directive 2010/63/EU). The experiment was approved by the local CEEA Vdl (certificate number 19-230113 for S. Tesseraud). The study was carried out at the PEAT INRAE Poultry Experimental Facility (https://doi.org/10.15454/1.5572326250887292E12) located at INRAE, Centre Val de Loire, Nouzilly, France, following the experimental procedures outlined in Conde-Aguilera et al., 2016a. The present study was carried out in parallel with the above-mentioned study, involving an additional number of chickens while the diets administered remained consistent across both studies (Supplemental Table 1).

Male broiler chicks Ross PM3 were conventionally reared under controlled conditions (lighting, environmental temperature and hygrometry) until reaching 24 d of age. After a standard one-week starter diet with 20 % crude protein and 1.19 % true digestible lysine (**Lys**), 40 birds were divided into 4 homogeneous groups (n = 10). These groups received diets classified as either deficient or sufficient in sulphur AA supplemented with varying levels of DL-Met (Met– and Met+, respectively) or DL-HMTBA (HMTBA– and HMTBA+, respectively). Diets based on corn, wheat, and soybean meal were formulated and served as a basis for the experimental diets. The 4 treatments differing in total sulphur AA (**TSAA**) levels (level-/level+) and Met source (DL-Met/HMTBA) were formulated into two extreme groups, constituting eight diets in total, to satisfy energy, crude protein and AA requirements from week 1 (d7 to d14) to week 3 (d21 to d24). For week 2 (d14 to d21), the birds received a diet resulting from blending the extreme diets formulated for weeks 1 and 3. The anticipated true digestible Lys content decreased from 1.16 to 1.02 % from week 1 to 3, in agreement with the recommendations of the breeder (Aviagen, 2009) and NRC (1994), except for sulphur AA. Deficient TSAA diets (Met– and HMTBA–) provided 33 % and 26 % digestible Met:Lys for week 1 and week 3, respectively, and 59 % and 52 % digestible TSAA:Lys for weeks 1 and 3, respectively. These supply levels were 45 % and 30 % lower than sufficient supply for Met:Lys and digestible TSAA:Lys, respectively.

Because the sulphur AA concentration in the diet may affect voluntary feed intake (Picard et al., 1993), the daily food allowance was slightly below the anticipated *ad libitum* intake capacity. The allowance was adjusted daily avoiding feed refusals, which (if any) were weighed to calculate actual ADFI. Animals readily consumed their daily feed allowance and feed refusals ranged from 0 to 7 % for the entire period, irrespective of the dietary treatment. Water was freely available throughout the study period.

### Sample collection and biochemical measurements

At 24 days of age, chickens were offered diets at least 2 h before to measure protein synthesis rates according to the well-established flooding dose method (Garlick et al., 1980; Sève et al., 1993; Tesseraud et al., 1996a; b). More precisely, a single intravenous injection of a Val flooding dose containing [^13^C]-Val was administered. Exactly 15 min after the stable tracer administration, a blood sample was collected from the occipital sinus. Subsequently, the chickens were slaughtered following stunning with bi-temporal electronarcosis and exsanguination.

Liver, breast (PM muscles), a part of thigh muscles, and a portion of jejunum were removed for sampling. Tissue subsamples were immediately frozen in liquid N and stored at –20°C for subsequent protein synthesis measurements, while another portion was stored at –80°C for proteolytic enzyme activity assessment (Le Naou et al., 2012), gene expression measurements and AA analyses.

The fractional synthesis rate of tissue protein (**FSR**, % of protein mass synthesized per day) was determined using the flooding dose procedure (Conde‑Aguilera et al., 2016c; Sève et al., 1993) with the following equation:

FSR(%)=(EB×100)/(EA×t)

where t is the L-[1-^13^C]Val incorporation time in minutes (i.e., time elapsed between the injection of the flooding Val dose and animal euthanasia), EA ( %) denotes the isotopic enrichment in the plasma-free pool, and EB ( %) signifies the isotopic enrichment in protein-bound tissues.

Protein and RNA concentrations were determined as previously described (Sève et al., 1993; Tesseraud et al., 1996a; b). The ribosomal capacity for protein synthesis, indicative of cellular protein synthesis potential, was calculated as the RNA:protein ratio (mg RNA/g protein), and the translational efficiency as the amount of protein synthesized per milligram of RNA per day. Furthermore, the absolute rates of protein synthesis (i.e. the total amounts of protein synthesized per day, in grams of protein per day) were also determined by multiplying FSR by protein mass (in grams) of the considered tissue (liver and the sum of 2 PM muscles).

Proteolytic activities of the proteasome (ATP-ubiquitin-dependent pathway) and of calpains (Ca^2+^-dependent pathway) were assayed in the PM muscles, thigh muscles, liver and jejunum, following established protocols (Conde‑Aguilera et al., 2016b; c; Le Naou et al., 2012). Briefly, samples were homogenized in a 20 mmol/L Tris-HCl buffer (pH = 7.2) containing DTT (1 mmol/L) and Triton X100 (1 %; Sigma, Steinheim, Germany). Subsequently, the mixtures were centrifuged for 15 min at 12,000 × g at 4°C. The chymotrypsin-like activity of the proteasome and calpain activities were measured by fluorimetry using succinylLeuLeuValTyr-7-amino-4-methylcoumarin as a substrate, with specific inhibitors clasto-lactacystin β-lactone (Calbiochem) and MDL-28170 (Biomol Research Laboratories, Plymouth, PA, USA) for proteasome and calpain, respectively. Protein concentration in supernatants was quantified by the Bradford method (Bradford, 1976).

### RNA isolation and RT-PCR

Total RNA was extracted using RNA Now (Biogentec) from 100 mg of tissue samples, following the manufacturer’s instructions. Subsequently, RNase-free DNase treatment was administered to eliminate DNA contamination. The purified RNA was reverse-transcribed using Super Script II RNase H Reverse transcriptase (Invitrogen) in the presence of random primers (Promega). Quantitative PCR assays were carried out in duplicate using the LightCycler 480 II apparatus (Roche). The primer sequences used for gene expression analysis encompassed genes associated with proteolysis (*FBXO32*, F-box only protein 32 also known as atrogin-1; *TRIM63*, RING-type E3 ubiquitin transferase also known as MuRF1; *UBB,* Ubiquitin*; PSMA1,* Proteasome subunit alpha type-1; *CAPN2*, Calpain 2; *CTSB,* Cathepsin B) and autophagy (*ATG12*, autophagy-related 12; *ATG4B*, autophagy-related 4b; *BNIP3*, Bcl-2/adenovirus E1B 19 kDa interacting protein 3; *MUL1*, mitochondrial ubiquitin ligase activator of NF-κB; *SQSTM1*, sequestosome 1; *UVRAG*, UV radiation resistance-associated gene protein). These primers were either specifically designed and validated, or previously used (Tesseraud et al., 2007; 2009), and are detailed in Supplemental Table 2. Gene expression levels were estimated on PCR efficiency and threshold cycle (Ct) deviation of an unknown sample versus a control, following established protocols (Pfaffl, 2001). To normalize gene expression data, the housekeeping gene *GAPDH* was utilized, which showed no diet effect.

### Free AA and peptide composition of the PM muscles

To determine peptide and free AA composition in the PM muscle, approximately 200 mg samples were ground using a domestic mincer and dispersed in high-purity water (1 g/2 ml) with a homogenizer (Ultra Turrax, Ika, Germany) for 230 seconds at 4°C. The homogenates were then centrifuged at 38,000 g for 1 h at 4°C. Proteins were removed from the supernatants by precipitation with trifluoroacetic acid at a concentration of 0.5 %, followed by centrifugation at 5,000 g for 20 minutes. To the supernatants from the last centrifugation, 100 ml aliquots were taken. These aliquots were stored at -80°C until AA analyses. They were analyzed by UPLC (Waters acquity ultra performance liquid chromatography system, Waters, Guyancourt, France) for free AA and peptide concentrations using the AccQtag Ultra method (Waters, Milford, MA, USA) (Eugenio et al., 2023).

### Statistical analyses

All the data of this experiment were statistically analysed using the GLM procedure of SAS software (SAS, 2004, SAS/STAT 9,1 user’s guide. SAS Institute Inc., Cary. NC) by two-way ANOVA using the individual animal as the experimental unit. The effects of Met level, also called TSAA level (level-/level+ whatever the Met source) and Met source (DL-Met/HMTBA), and their interaction were included in the statistical model with an alpha value of 5 %. When interactions between the main factors were significant, the means of each of the four treatment combinations were compared by one-way ANOVA, and an LSD post hoc test was used to identify the significant difference (SAS, 2004). Results are reported as least-square means.

## Results

### Animal and tissue characteristics

The sulphur AA supply significantly affected performance, resulting in lower final body weight (**BW**), average daily gain (**ADG**) and higher feed conversion ratio (**FCR**) values in chickens receiving the TSAA-deficient diets (Met– and HMTBA–; *P* < 0.01; Table 1). The source of Met showed no discernible impact on any of these performance parameters. Consequently, animals consuming TSAA-deficient diets exhibited lower weights in the PM muscles (*P* < 0.001; Table 1), regardless of the type of Met ingested, with no changes observed in the weight of liver. The protein content of the jejunum was lower (*P* < 0.05) in animals receiving TSAA-deficient diets, regardless of the Met source. The protein content of other tissues and organs (liver, PM and thigh muscles) remained unaffected. The type of Met used did not affect either the weight or the protein content of the various tissues analyzed. Additionally, we did not observe any interaction between the level of Met and the source in relation to these parameters.

**Table 1:** Performance and tissue characteristics of broilers offered a diet deficient (Met– and HMTBA–) or sufficient (Met+ and HMTBA+) in sulphur AA from 7 to 24 d of age^1^.

	Treatment					*P*-value		
Item	Met–	Met+	HMTBA–	HMTBA+	RSD	Met level	Met source	LxS
Initial BW (g)	150	148	151	153	9	0.96	0.42	0.48
Final BW (g)	1 078	1 102	1 067	1 105	31	<0.01	0.70	0.51
ADFI (g/d)	77.0	80.6	78.5	78.9	NA	NA	NA	NA
ADG (g/d)	54.6	56.1	53.9	56.0	1.9	<0.01	0.56	0.67
FCR (g/g)	1.45	1.40	1.46	1.41	0.02	<0.01	0.58	0.71
Tissue weight at d24 (g)								
*Pectoralis major* muscles^2^	137	167	132	160	16	<0.001	0.27	0.79
Liver	39.6	42.3	39.9	39.3	3.6	0.35	0.25	0.16
Tissue protein content (mg/g)								
*Pectoralis major* muscles	89.3	92.8	95.6	94.9	7.9	0.58	0.11	0.42
Thigh muscles	101	101	100	105	8.0	0.28	0.66	0.46
Liver	116	116	114	117	8.0	0.52	0.96	0.46
Jejunum	72.5	79.2	75.6	78.1	5.8	0.02	0.60	0.27

1 Least-squares means, n = 10. No statistical analysis was performed for daily feed intake which was adjusted to be equalized between dietary treatments. LxS, interaction between Met level and Met source; NA, not applicable; *P*, probability for a treatment effect; RSD, residual SD.

2 Weight considering 2 *Pectoralis major* muscles.

### Tissue protein synthesis

Both the level of Met and the Met source had a significant impact on the fractional synthesis rate (FSR) of the liver (Table 2). The FSR was higher when animals were fed TSAA-deficient diets (Met– and HMTBA–; *P* < 0.01), as well it was registered higher rate in the groups receiving HMTBA into the diet compared to Met diets (*P* = 0.04). The FSR were also greater in the jejunum of chickens receiving TSAA-deficient diets (*P* < 0.01), but the effect of the Met source did not reach statistical significance in this tissue (*P* = 0.12). Neither muscles (PM and thigh muscles) were consistently affected by the TSAA deficiency, except for the thigh muscle in the group of animals deficient in HMTBA (HMTBA–), which showed a higher value of FSR, due to an interaction between the source and level of Met.

**Table 2:** Tissue protein synthesis of broilers offered a diet deficient (Met– and HMTBA–) or sufficient (Met+ and HMTBA+) in sulphur AA from 7 to 24 d of age^1^.

	Treatment					*P*-value		
Item	Met–	Met+	HMTBA–	HMTBA+	RSD	Met level	Met source	LxS
Fractional synthesis rate	*%/d*							
*Pectoralis major* muscles	21.9	19.9	22.0	20.4	3.7	0.14	0.80	0.87
Thigh muscles	21.7^a^	21.5^a^	24.0^b^	20.1^a^	2.4	0.01	0.55	0.02
Liver	95.6	83.1	102.0	93.6	12.0	0.01	0.04	0.60
Jejunum	112	101	115	106	8	<0.01	0.12	0.67
Absolute protein synthesis	*g/d*							
*Pectoralis major* muscles^2^	2.66	3.08	2.75	3.07	0.51	0.03	0.82	0.77
Thigh muscles	–	–	–	–	–	–	–	–
Liver	4.34	4.03	4.61	4.27	0.48	0.04	0.11	0.92
Jejunum	–	–	–	–	–	–	–	–
Translational efficiency of protein synthesis	*mg protein/mg RNA/d*							
*Pectoralis major* muscles	17.2	14.1	17.0	15.6	3.5	0.05	0.54	0.43
Thigh muscles	15.1	14.7	15.7	14.2	1.7	0.09	0.95	0.29
Liver	14.6	14.1	16.2	15.5	2.4	0.49	0.06	0.87
Jejunum	13.8	14.2	14.5	14.3	2.8	0.89	0.68	0.75
RNA:protein ratio	*mg RNA/g protein*							
*Pectoralis major* muscles	13.0	14.3	13.4	13.3	2.0	0.34	0.64	0.25
Thigh muscles	14.5	14.6	15.3	14.2	1.2	0.20	0.55	0.14
Liver	68.1	59.4	63.9	61.1	11.7	0.13	0.73	0.43
Jejunum	83.2	72.1	81.5	77.7	14.5	0.12	0.68	0.44

1 Least-squares means, n = 10. LxS, interaction between Met level and Met source; –, not determined in the absence of total jejunum protein content; *P*, probability for a treatment effect; RSD, residual SD.

a–b When there is a significant interaction between Met level and source, values within a row without a common letter differ by pairwise comparisons, *P* < 0.05.

2 Absolute protein synthesis considering 2 *Pectoralis major* muscles; –, not determined because the tissue was not removed in its entirety and was not weighed.

On the other hand, absolute protein synthesis was lower in the PM muscle of TSAA-deficient chickens (*P* = 0.03), regardless of the Met source. In contrast, in the liver, this absolute protein synthesis was significantly higher in TSAA-deficient animals (*P* = 0.04).

The translational efficiency of protein synthesis, assessed as mg of protein per mg of RNA per day, exhibited higher levels in the PM muscles (*P* = 0.05) and tended towards significance in the thigh muscles (*P* = 0.09) within the TSAA-deficient group, irrespective of the Met source. Interestingly, there was a notable trend towards increased translational efficiency of protein synthesis in the liver when HMTBA was included in the diets (*P* = 0.06). In contrast, no significant changes in this translational efficiency were observed in the remaining tissues (*i.e.* PM and thigh muscles, and jejunum) according to the Met source. Additionally, the RNA/protein ratio remained unaffected across all tissues studied under the given treatments to the chickens.

### Tissue proteolytic activities and muscle gene expression

Regarding the proteolytic activity of enzymes, proteasome activity was significantly higher in all tissues studied in TSAA-deficient diets (Met– and HMTBA–; Table 3; *P* < 0.01), regardless of the Met source. There was no effect of Met source, except in the liver, which exhibited higher proteasome proteolytic activity when animals received HMTBA as the source, compared to DL-Met (*P* < 0.05). On the other hand, calpain activity was significantly increased in the jejunum (*P* < 0.0001) and tended to be higher in the thigh muscles (*P* = 0.08) and when chickens received TSAA-deficient treatments, with a substantial increase observed when HMTBA was incorporated into the diet (*P* < 0.01) compared to DL-Met. The liver also showed higher calpain activity under this same HMTBA source compared to animals receiving DL-Met (*P* = 0.01). Importantly, there was no interaction between the level and source of Met.

**Table 3:** Tissue proteolytic enzyme activities of broilers offered a diet deficient (Met– and HMTBA–) or sufficient (Met+ and HMTBA+) in sulphur AA from 7 to 24 d of age^1^.

	Treatment					*P*-value		
Item	Met–	Met+	HMTBA–	HMTBA+	RSD	Met level	Met source	LxS
Proteasome activity								
*Pectoralis major* muscles	13.2	10.2	13.2	10.8	1.4	<0.001	0.47	0.48
Thigh muscles	10.2	9.1	10.1	8.7	1.4	0.01	0.63	0.75
Liver	113	85	123	103	19	<0.001	0.04	0.60
Jejunum	76.5	58.7	84.6	54.1	19.9	<0.01	0.81	0.39
Calpain activity								
*Pectoralis major* muscles	3.68	3.66	3.64	3.68	0.87	0.97	0.99	0.91
Thigh muscles	7.12	6.11	7.17	6.88	1.03	0.08	0.26	0.33
Liver	216	209	234	235	22	0.70	0.01	0.59
Jejunum	348	261	395	142	28	<0.0001	<0.01	0.11

1 Least-squares means, n = 10. Activity enzymes are expressed as (relative fluorescence units) · min^-1^ · (g protein)^-1^. LxS, interaction between Met level and Met source; *P*, probability for treatment effect; RSD, residual SD.

In terms of the expression of proteolysis and autophagy-related genes in the PM muscle, most of these genes were influenced by the level of Met in the diet (Table 4). Expression was higher in TSAA-deficient diets for *TRIM63, UBB, PSMA1, ATG12, ATG4B, UVRAG, BNIP3*, and *MUL1* (*P* < 0.05) and conversely lower for *FBXO32*. The Met source only influenced increasing the expression of the *BNIP3* gene (*P* = 0.02), and showed a trend indicating a potential decrease in the expression of *SQSTM1* (*P* = 0.09), when HMTBA was incorporated into the chickens' diet. No interaction was observed between the level and source of Met.

**Table 4:** Proteolysis- and autophagy-related gene expression levels in the *pectoralis major* muscles of broilers offered a diet deficient (Met– and HMTBA–) or sufficient (Met+ and HMTBA+) in sulphur AA from 7 to 24 d of age^1^.

	Treatment					*P*-value		
Item	Met–	Met+	HMTBA–	HMTBA+	RSD	Met level	Met source	LxS
*FBXO32*	1.03	1.73	1.03	1.58	0.63	0.03	0.77	0.78
*TRIM63*	1.07	0.76	0.97	0.69	0.25	< 0.001	0.12	0.81
*UBB*	0.86	0.62	0.76	0.67	0.28	0.01	0.66	0.24
*PSMA1*	0.92	0.74	1.02	0.78	0.21	< 0.001	0.16	0.47
*CAPN2*	1.72	1.46	1.73	1.51	0.38	0.24	0.89	0.92
*CTSB*	1.01	1.10	1.07	1.04	0.19	0.67	0.99	0.36
*ATG12*	1.09	0.87	1.32	0.90	0.37	< 0.01	0.26	0.38
*ATG4b*	0.84	0.72	1.02	0.74	0.25	< 0.01	0.10	0.17
*UVRAG*	0.90	0.84	1.03	0.86	0.17	0.02	0.14	0.26
*SQSTM1*	1.33	1.27	1.19	1.14	0.20	0.50	0.09	0.96
*BNIP3*	0.84	0.69	0.98	0.74	0.19	< 0.001	0.02	0.23
*MUL1*	1.28	1.01	1.35	1.01	0.24	< 0.001	0.66	0.71

1 Least-squares means, n = 9-10. The mRNA levels of the target genes were measured by quantitative real time polymerase chain reaction (RT-qPCR). Expression values (arbitrary units) were corrected for *GAPDH* mRNA, which did not differ significantly according to the group. LxS, interaction between Met level and Met source; *P*, probability for treatment effect; RSD, residual SD. Target genes: *FBXO32*, F-box only protein 32 also known as atrogin-1; *TRIM63*, RING-type E3 ubiquitin transferase also known as MuRF1; *UBB,* Ubiquitin; *PSMA1*, Proteasome component C2 / Proteasome subunit alpha type-1; *CAPN2*, Calpain 2; *CTSB*, Cathepsin B; *ATG12*, autophagy-related 12; *ATG4b*, autophagy-related 4b; *UVRAG*, UV radiation resistance-associated gene protein; *SQSTM1*, sequestosome 1; *BNIP3*, Bcl-2/adenovirus E1B 19 kDa-interacting protein 3; *MUL1*, mitochondrial ubiquitin ligase activator of NF-κB

### Muscle peptide and free AA composition

The deficiency in Met had a significant effect on the content of peptides and free AA related to the antioxidant activity of the PM muscle, resulting in decreased levels of anserine, β-alanine, and balenine, while carnosine, histidine (**His**), and taurine were increased (Table 5; *P* < 0.05), regardless of the source of Met. Only the reduction in balenine was significant in chickens fed with HMTBA compared to DL-Met. Moreover, the groups of animals fed with TSAA-deficient diets exhibited significantly increased muscle contents of free glycine, Lys, serine, and threonine (**Thr**) (*P* < 0.05; a tendency was observed for free glutamate, *P* = 0.09) and conversely decreased contents of free proline, whatever the type of Met used.

**Table 5:** Free AA and peptide composition of the *pectoralis major* muscles of broilers offered a diet deficient (Met– and HMTBA–) or sufficient (Met+ and HMTBA+) in sulphur AA from 7 to 24 d of age^1^.

	Treatment					*P*-value		
Item	Met–	Met+	HMTBA–	HMTBA+	RSD	Met level	Met Source	LxS
Antioxidant-related								
1-Methylhistidine^2^	0.52	0.52	0.55	0.49	0.07	0.31	0.90	0.29
3-Methylhistidine^2^	0.63	0.54	0.52	0.54	0.12	0.43	0.27	0.26
Alanine	12.0	13.1	10.7	13.5	2.59	0.08	0.66	0.40
Anserine	335	570	367	542	67.6	<0.01	0.95	0.29
β-Alanine	5.56	9.39	4.55	9.21	3.61	0.01	0.68	0.78
Balenine	2.77	6.36	1.87	5.68	0.73	<0.01	0.02	0.72
Carnosine	456	168	436	212	45.2	<0.01	0.52	0.09
Histidine	1.86	0.41	1.69	0.45	0.27	<0.01	0.54	0.31
Ornithine	1.51	0.92	1.45	1.64	0.83	0.55	0.34	0.25
Taurine	12.66	8.12	12.53	9.19	4.64	0.04	0.80	0.75
Other functions								
Arginine	3.33	2.56	2.88	1.86	1.35	0.12	0.30	0.82
Aspartate	2.01	1.77	2.14	2.19	0.58	0.53	0.26	0.69
Glutamate	13.2	10.2	11.9	11.8	2.20	0.09	0.85	0.11
Glycine	15.95	6.46	12.30	5.70	2.72	<0.01	0.06	0.20
Hydroxyproline	2.85	3.37	6.93	3.26	3.39	0.26	0.16	0.14
Isoleucine	1.64^b^	1.22^a^	1.36^a^	1.39^a^	0.27	0.09	0.57	0.05
Leucine	2.81^b^	2.09^a^	2.31^a^	2.45^a^	0.46	0.13	0.71	0.03
Lysine	14.12	6.91	15.67	7.59	3.42	<0.01	0.46	0.77
Methionine	0.64	0.63	0.53	0.57	0.19	0.83	0.29	0.74
Phenylalanine	2.04^b^	1.65^a^	1.60^a^	1.74^a^	0.29	0.29	0.15	0.03
Proline	2.98	5.22	2.39	4.97	1.59	<0.01	0.52	0.79
Serine	15.3	10.7	12.4	11.7	2.61	0.02	0.36	0.08
Threonine	10.63	4.42	9.89	5.70	1.74	<0.01	0.71	0.16
Valine	2.51^b^	2.02^a^	2.09^a^	2.23^a^	0.37	0.25	0.50	0.05

1 Least-squares means, n = 10. Composition was expressed as µmol/100 g lyophilized muscle*.* HMTBA, DL-2-hydroxy-4-methylthiobutyric acid; LxS, interaction between Met level and Met source; *P*, probability for a treatment effect; RSD, residual SD.

2 1-Methylhistidine also known as tau-methylhistidine; 3-Methylhistidine also known as pi-methylhistidine

a–b When there is a significant interaction between Met level and source, values within a row without a common letter differ by pairwise comparisons, *P* < 0.05.

There was no effect of Met source, except for glycine content, which tended to be higher in the groups receiving DL-Met versus HMTBA. Due to an interaction between the level and source of Met, we found that the contents of free isoleucine, leucine, phenylalanine, and valine in the muscle were significantly higher only in the Met– group compared to the other three dietary treatments.

## Discussion

This study explores the impact of Met supply on chicken tissue protein turnover, extending our previous findings on the subject. We previously reported that ADG and FCR measured between 7 and 21 days of age, were significantly impaired in TSAA-deficient chickens compared to TSAA-sufficient chickens (3-4 %, Conde-Aguilera et al., 2016a). In another subgroup of animals from the same experiment with a slightly broader age (7-24 days, present study), we found a deterioration of the same magnitude for ADG and FCR regardless of the Met source. The use of HMTBA or DL-Met did not affect performance irrespective of Met level, as anticipated (Conde-Aguilera et al., 2016a), indicating that the source of Met may be less critical than its overall availability. To further understand the underlying mechanisms, we focused on tissue protein turnover by quantifying *in vivo* protein synthesis rates (^13^C-Valine incorporation) and measuring several parameters related to proteolysis or autophagy. The lower chicken performance induced by a deficient supply in TSAA was accompanied by a reduction in the weight and amount of protein synthesized in PM muscles at 24 days of age. In contrast, liver weight was unaffected by TSAA deficiency and the amount of protein synthesized in the liver was even higher in the TSAA– chickens compared to TSAA-sufficient chickens, indicating different responses according to the tissue. The effects were less contrasted when proteolytic activities were considered, particularly for the ubiquitin-dependent proteasome system. Proteasome activities were thus higher in TSAA-deficient chickens compared to TSAA-sufficient chickens, regardless of the tissue considered and the Met source. While Met deficiency alters PM muscle development and tissue protein turnover, different Met sources have little effect.

### Divergent changes in tissue protein synthesis due to Met deficiency

The fractional synthesis rate of tissue protein (FSR in %/d) was determined using the flooding dose procedure in 24 d-old chickens. We found that protein synthesis rates varied considerably between different tissues, with FSR presented in decreasing rank order: 103 %/d (jejunum), 88 %/d (liver), and 20-21 %/d (skeletal muscle) in control chickens (i.e. diets sufficient in TSAA). These values align with the literature for both chickens (Tesseraud et al., 1996a; b) and pigs (Sève et al., 1993; Conde-Aguilera et al., 2016b; c), despite a lower FSR in liver and skeletal muscle in pigs compared to chickens.

The results obtained here on protein synthesis according to Met supply seem quite complex. Firstly, they may depend on how synthesis rates are expressed, for example in PM muscles. While the amount of proteins synthesized in PM muscles was significantly lower (-12 %) in TSAA-deficient chickens compared to TSAA-sufficient chickens, FSR were not significantly altered, because the protein mass in PM muscles was itself concomitantly lower (-19 %). Different responses of muscle absolute synthesis rate and FSR have been previously reported in case of Lys deficiency due to the significant reduction in the protein mass in PM muscles (Tesseraud et al., 1996b; 2001). Another major factor explaining the variation between results is the tissue considered, with differences in response between PM muscles and liver for example under Met deficiency (present study) and Lys deficiency (Tesseraud et al., 1996b). Similar findings have been found with differences in response between muscles of different types under Met deficiency (here) and Lys deficiency (Tesseraud et al., 1996a). The present study indicates that Met deficiency did not change FSR in PM muscles, increased FSR in thigh muscles but only in the group of chickens HMTBA– (source x level of Met interaction), and increased FSR in the liver and jejunum regardless of Met source. These results conflicts with those of two studies performed in piglets (Conde-Aguilera et al., 2016b; c), in which Met deficiency either decreased FSR (*Longissimus* muscle) or did not change FSR (*Rhomboideus* and *Semitendinous* muscles; jejunum, ileum and liver). In the latter cases, an explanation would be related to the species rather than the level of deficiency, which was similar (sulphur AA deficiency of around -30 %), perhaps partly because Met is the first limiting AA in chickens and not in pigs.

When comparing results between studies in the literature, the specific AA presenting a deficiency is also a crucial factor to consider (Hocquette et al., 2007; Pacheco et al., 2018; Tesseraud et al., 2011). This is particularly true when comparing Met and Lys deficiencies. Whereas Lys is used almost entirely for body protein synthesis and does not take part in other metabolic processes (except as a precursor of carnitine), Met is less efficiently retained into body due to its multiple roles in metabolism and cell functions, and as carrier of 1C (methyl) groups. A relatively more moderate Lys deficiency (-23 %, Tesseraud et al., 1996b) than that used in the present study for sulphur-containing AA (-30 %, or -43 % if only Met and not the sum of Met + Cys is considered) decreased chicken daily weight gain of -50 % *vs*. -3 % with Lys and Met deficiencies, respectively. Moreover, it reduced tissue weights more strongly than Met (-65 % *vs*. -18 % with Lys and Met deficiencies, respectively in PM muscles; -47 % *vs*. -2.5 % with Lys and Met deficiencies, respectively in liver). Nevertheless, irrespective of specific AA deficiency, while PM muscles are highly sensitive to AA deficiency, the liver may be protected from atrophy, perhaps due to its vital functions or favourable localisation for nutrient access compared to peripheral tissues such as muscles.

The mechanisms regulating synthesis seem to differ considerably depending on the tissue or species considered and/or on which AA is deficient. For example, the translational efficiency of protein synthesis was here higher in the skeletal muscles of TSAA-deficient chickens than in TSAA-sufficient chickens, at least for PM muscles (trend in thigh muscles), but unchanged by Met supply in splanchnic tissues (liver and jejunum). Conversely, it was not modified by Lys deficiency irrespective of tissue (PM muscles and liver, Tesseraud et al., 1996b). Nor do the results of the present study agree with those found in piglets, which even show a decrease by Met deficiency in the translational efficiency of protein synthesis in skeletal muscles (at least in *Longissimus* and *Semitendinous* muscles; not significant in *Rhomboideus* muscles, Conde-Aguilera et al., 2016b) without any change in intestine and liver (Conde-Aguilera et al., 2016c). A similar controversy exists for ribosomal capacity, but this mainly concerns skeletal muscle, where deficiency-related differences depend on the AA considered. Our study shows that ribosomal capacity for muscle protein synthesis, i.e. the RNA:protein ratio, was not significantly affected by the level of Met supply, meaning that the protein synthesis machinery was not really altered in the tissues studied (only numerically higher values in the TSAA-deficient groups in liver and jejunum, independently of source). These findings are consistent between species (RNA:protein ratio was not affected by Met supply in piglets, Conde-Aguilera et al., 2016b; c), but contrast with the great increase in ribosomal capacity due to Lys deficiency in *Sartorius* and especially PM muscles (Tesseraud et al., 1996b; 2001), which could be linked to a delay in development acting on capacities for protein synthesis and FSR as discussed previously by Tesseraud et al., 1996b.

### Increased proteolysis and autophagy markers in response to Met deficiency

The chymotrypsin-like activity of the proteasome was clearly higher in TSAA-deficient birds than in TSAA-sufficient groups, for all tissue studied: +43 % (jejunum); +33 and +19 % for Met and HMTBA groups, respectively (liver); +26 % (PM muscles); +14 % (thigh muscles). The ubiquitin-proteasome proteolytic pathway, which is a major route of protein degradation, was therefore particularly altered by Met level. Calpain proteinases represent another of the three major proteolytic systems involved in tissue proteolysis. Whereas calpain activity was unchanged by the level of Met supply in PM muscle and liver, it exhibited higher values in TSAA-deficient birds than in TSAA-sufficient groups in jejunum (+33 and +178 % depending on the Met source, respectively), and tended to be higher in TSAA-deficient groups than in TSAA-sufficient groups in the thigh muscles. Surprisingly, absolutely no Met deficiency-related changes were observed in the activities of these 2 proteolytic systems in piglets, regardless of the tissue considered (liver, jejunum, ileum and 3 types of muscle, Conde-Aguilera et al., 2016b; c), suggesting that protein breakdown was not affected in this species, unlike chickens. To further investigate the regulation of proteolysis, we focussed on PM muscles by determining the expression of genes involved in this process. Gene expression was higher in TSAA-deficient birds than in TSAA-sufficient groups for *TRIM63* (E3 ubiquitin ligase), *Ubiquitin* and *PSMA1* (proteasome subunit alpha type-1), which are involved in the ubiquitin-proteasome proteolytic pathway, but increased for *FBXO32* (another E3 ubiquitin ligase), or unchanged for *CAPN2* (Calpain 2) and *CTSB* (Cathepsin B), involved in the calpain and lysosomal proteolytic pathways respectively. *TRIM63* and *FBXO32* belong to two families of E3 ubiquitin ligases using different proteins (RING and F-box proteins, respectively) to target protein substrates for ubiquitination, with these substrates subsequently being selected by the proteasome (Hughes et al., 2023). The two E3 ubiquitin ligases are regulated by various lifestyle factors, including nutritional and antioxidant factors (Rom and Reznick, 2016), but sometimes not in the same way, as already shown in chickens in a model using cyclical nutritional treatments with diets varying in protein and/or energy content (Boussaid-Om Ezzine et al., 2012). They may play different roles in the control of muscle mass. *TRIM63* is involved in the degradation of myofibrillar proteins (myosin heavy and light chains and other myofibrillar proteins), and might therefore be associated with muscle proteolysis, whereas changes in *FBXO32* do not appear to be always correlated with muscle proteolysis rates (Attaix and Baracos, 2010).

In addition, Met deficiency also resulted in higher expression of genes involved in autophagy, such as *ATG12, ATG4B, UVRAG, BNIP3* and *MUL1*, but not *SQSTM1*. Some of these results are in line with the literature, with occasional differences depending on the gene studied, the species of interest or the age of the animal. For example, it has been shown that Met deficiency induces higher mRNA levels of *TRIM63* (also called *MuRF1*) in chickens (days 10 and 21, but not on day 35; Zeitz et al., 2019), this gene being invariant in trout (Belghit et al., 2014), while it increases expression of the *FBXO32, ATG12, ATG4B, UVRAG, MUL1* and *BNIP3* genes in trout (Belghit et al., 2014). The effects may also depend on the variation in Met supply, and in particular on the comparison between a level meeting the animals' needs and a deficient level, or on the contrary, an intake that is well above requirements, as shown in the studies by Zeitz et al. (2019) and Belghit et al. (2014).

Muscle growth is determined by the balance between protein synthesis and proteolysis. It is difficult to know whether muscle protein synthesis is less affected by Met deficiency than degradation, especially if the approaches used are only indirect, targeting gene expression levels and/or activation of signaling pathways controlling protein turnover (Wen et al., 2014 and Zeitz et al., 2019 in chickens; Belghit et al., 2014 in trout). Here, the lower PM weights in TSAA-deficient birds appeared largely explained by higher proteolytic enzyme activity associated with higher mRNA levels of proteolysis- and autophagy-related genes, without any decrease in muscle FSR. Similarly, it was previously observed that Lys restriction increased the rate of protein degradation in PM muscles, while FSR was not reduced (Tesseraud et al., 1996; 2001 and 2009). If we consider the quantity of protein synthesized per day and not in terms of FSR ( %/d), absolute protein synthesis is lower regardless of the AA studied. The increased concentration we reported in the present study for several free AAs, including His, Lys, and Thr, but not Met, is consistent with the lower amount of protein synthesized in PM muscles of birds receiving a limiting Met supply. Nevertheless, our work also suggests that muscle protein turnover is differently affected by AA deficiency in chickens and piglets, for which Met deficiency led to invariant proteolytic activities and lower FSR in the glycolytic *Longissimus* muscle (Conde-Aguilera et al., 2016b). Given Met's role in tissue antioxidant activity, we also studied the composition of PM muscle in terms of peptides and free AAs linked to antioxidant activity. Whereas free contents of β-alanine, anserine and balenine (2 dipeptides produced from β-alanine and methylhistidine) of PM in TSAA-deficient birds were lower than in the TSAA-sufficient groups, free contents of His, carnosine (a dipeptide produced from β-alanine and His) and taurine were higher. Besides its pH buffering capacity, carnosine has antioxidant properties providing an important defense mechanism against oxidative stress in skeletal muscles (Boldyrev et al., 2013), as do the other antioxidant molecules (i.e. His and taurine) for which increased content was also observed in PM of TSAA-deficient animals. These antioxidant mechanisms could have been initiated for defense against oxidative stress since the antioxidant status may have been compromised by lower muscle glutathione concentrations due to the limiting sulphur AA supply (Conde-Aguilera et al., 2016b). Moreover, it is interesting to see the inverse evolution of anserine and carnosine in Met deficiency. Both dipeptides have similar antioxidant properties, although anserine is more important in birds than in terrestrial mammals. However, they differ in the methylation of His. The methylated form for anserine may explain its lower concentrations in Met (methyl donor) deficient groups, as also observed for balenine, another methylhistidine-containing dipeptide. Therefore, the chickens seem to be able the antioxidant properties of these His-containing dipeptides, but favor the non-methylated form when Met is limiting.

### Similar effects across different Met sources

Our study demonstrated that changing the dietary level of Met or HMTBA results in similar changes in growth performance, tissue weight and protein content, without any effect of Met source. Because the sulphur AA concentration in the diet may affect voluntary feed intake (Picard et al., 1993), the diet was distributed slightly below the anticipated *ad libitum* intake capacity. In these conditions, daily feed intake was strictly the same for the 4 groups of chickens, and therefore unchanged according to Met source. Furthermore, assuming that intestinal absorption of the two Met sources is similar as is also the relative *in vivo* conversion of D-MET, D-HMTBA and L-HMTBA to L-MET as indicated by pool sizes of Met, tRNA^Met^ and tRNA^Cys^ (Met and Cys acylated to tRNA) (Barnes et al., 1995), both Met and HMTBA may be efficiently used by the chicken. We also observed no Met source-related differences in muscle protein synthesis, regardless of muscle type. Accordingly, it has been shown that dietary Met and HMTBA can be used with similar efficacy to support skeletal muscle protein accretion and rates of protein synthesis when feed intake is equalized (Barnes et al., 1995). Moreover, proteolytic activities were unaffected by Met source in the present study, and except for *BNIP3*, there were no differences in the mRNA levels of proteasomal and autophagy-related genes in PM muscles, as previously found for *SQSTM1, TRIM63, FBXO32, ATG5, ATG9A* and *BECN1 (Beclin 1)* in breast muscle of broilers at days 10, 21 and 35 (Zeitz et al., 2019). In the latter study, the activation of signaling pathways regulating protein turnover was also unaffected by Met source.

The differences related to Met source observed in protein metabolism were mainly in splanchnic tissues, particularly in the liver, as in the jejunum protein synthesis and proteasome activity remained unchanged, with only calpain activity being affected by Met source. In the liver, various protein metabolism parameters differed by Met source, with higher values in HMTBA groups compared to Met groups for FSR, proteasome and calpain activities. The mechanisms underlying these differences are not well understood. Regarding protein synthesis, the changes were not related to alterations in the protein synthesis machinery, as the RNA/protein ratio remained unchanged. However, there was a tendency for a greater translational efficiency in protein synthesis in HMTBA groups compared to Met groups. Studies comparing Met sources have examined Met incorporation in various tissues, including the liver, using labelled Met with ^14^C or ^35^S. No consistent difference was found in the incorporation of radiolabeled Met sources into liver protein for L-Met, DL-Met, and HMTBA in chickens (see Becquet et al., 2023 for a review). Further studies are needed to better understand the metabolism of sulphur AA and clarify the metabolism of different Met sources using sulphur-labelled molecules, as recently proposed (Lalande et al., 2025).

## Conclusions

A Met deficiency greatly alters chicken growth, PM muscle development, protein synthesis and degradation with effects specific to different tissues, while only minor differences were observed between the two Met sources. Our results also indicate that, when adequately supplied, both DL-HMTBA and DL-Met effectively support protein synthesis and accretion in muscle without differentially affecting protein breakdown. These findings also suggest that Met supply and its effects on protein metabolism vary between species and tissues, highlighting the complexity of the regulatory mechanisms governing protein synthesis and proteolysis in response to AA deficiencies.

## Funding

This research was supported in part by Rhodimet Research Grant (Adisseo France S.A.S., Antony, France) and INRAE.

## Data availability

The datasets produced and/or analyzed in this paper are available from the corresponding author upon request.

## Declaration of competing interest

The authors declare that they have no known competing financial interests or personal relationships that could have appeared to influence the work reported in this paper.

## Appendix Supplementary materials

Image, application 1
