# Supplementary material for: Effects of a methionine deficiency on chicken tissue protein turnover: comparative analysis of methionine source
Source: Poult Sci. 2025 Jun 7;104(9):105410. doi: 10.1016/j.psj.2025.105410 (PMC12210298; doi:10.1016/j.psj.2025.105410)
Supplement: Supplementary file 1 [file mmc1.docx]

**Supplemental Table 1** Ingredients and nutrient composition of the basal diets (as-fed basis)^1^

|  | week 1 (d7-14) | | | |  | week 3 (d21-25) | | | |
| --- | --- | --- | --- | --- | --- | --- | --- | --- | --- |
| Item (%) | Met– | Met+ | HMTBA– | HMTBA+ |  | Met– | Met+ | HMTBA– | HMTBA+ |
| Ingredients |  |  |  |  |  |  |  |  |  |
| Corn | 36.08 | 35.78 | 36.06 | 35.71 |  | 41.61 | 41.38 | 41.60 | 41.33 |
| Soybean meal | 36.24 | 36.24 | 36.24 | 36.24 |  | 27.33 | 27.33 | 27.33 | 27.33 |
| Wheat | 20.00 | 20.00 | 20.00 | 20.00 |  | 25.00 | 25.00 | 25.00 | 25.00 |
| Soybean oil | 3.85 | 3.85 | 3.85 | 3.85 |  | 2.44 | 2.44 | 2.44 | 2.44 |
| DL-Methionine | 0.10 | 0.39 | - | - |  | 0.02 | 0.24 | - | - |
| DL-HMTBA^2^ | - | - | 0.12 | 0.47 |  | - | - | 0.02 | 0.29 |
| L-Lysine HCl | 0.14 | 0.14 | 0.14 | 0.14 |  | 0.22 | 0.22 | 0.22 | 0.22 |
| L-Threonine | 0.00 | 0.00 | 0.00 | 0.00 |  | 0.01 | 0.01 | 0.01 | 0.01 |
| Salt | 0.30 | 0.30 | 0.30 | 0.30 |  | 0.30 | 0.30 | 0.30 | 0.30 |
| Calcium carbonate | 1.08 | 1.08 | 1.08 | 1.08 |  | 1.17 | 1.17 | 1.17 | 1.17 |
| Dicalcium phosphate | 1.66 | 1.66 | 1.66 | 1.66 |  | 1.36 | 1.36 | 1.36 | 1.36 |
| Premix^3^ | 0.50 | 0.50 | 0.50 | 0.50 |  | 0.50 | 0.50 | 0.50 | 0.50 |
| Monensin | 0.05 | 0.05 | 0.05 | 0.05 |  | 0.05 | 0.05 | 0.05 | 0.05 |
| Analyzed composition^4^ |  |  |  |  |  |  |  |  |  |
| Crude protein | 23.2 | 22.8 | 23.0 | 23.5 |  | 20.5 | 20.5 | 20.2 | 20.2 |
| Crude fat | 6.2 | 6.0 | 6.0 | 5.9 |  | 3.8 | 3.8 | 3.7 | 3.8 |
| Starch | 38.4 | 37.7 | 38.6 | 38.4 |  | 46.5 | 45.0 | 46.0 | 45.9 |
| Ash | 5.9 | 5.8 | 5.7 | 6.0 |  | 5.4 | 5.4 | 5.3 | 5.4 |
| Gross energy (Mcal/kg) | 4.1 | 4.1 | 4.1 | 4.1 |  | 4.1 | 4.1 | 4.1 | 4.1 |
| Lys | 1.35 | 1.30 | 1.34 | 1.36 |  | 1.2 | 1.18 | 1.23 | 1.24 |
| Met^2^ | 0.38 | 0.67 | 0.41 | 0.69 |  | 0.27 | 0.47 | 0.28 | 0.50 |
| TSAA^5^ | 0.82 | 1.10 | 0.86 | 1.14 |  | 0.67 | 0.90 | 0.71 | 0.92 |
| Thr | 0.87 | 0.88 | 0.88 | 0.88 |  | 0.77 | 0.75 | 0.79 | 0.78 |
| Calculated composition^4,6^ |  |  |  |  |  |  |  |  |  |
| AME_n_ (Mcal/kg) | 3.08 | 3.04 | 3.07 | 3.07 |  | 3.07 | 3.07 | 3.06 | 3.05 |
| Digestible Lys | 11.6 | 11.6 | 11.6 | 11.6 |  | 10.2 | 10.2 | 10.2 | 10.2 |
| Digestible Met:Lys^7^ | 33 | 58 | 33 | 58 |  | 26 | 48 | 26 | 48 |
| Digestible TSAA:Lys^7^ | 59 | 84 | 59 | 84 |  | 52 | 75 | 52 | 75 |

^1^The 8 diets differed in total sulphur AA (**TSAA**) contents by 2 sources of adding Met on the one hand and in energy, crude protein and AA contents on the other hand to account for the change in nutrient requirements between week 1 and week 3. These diets were blended proportionally for week 2. Met–, diet deficient in Met by dl-Met supplementation; Met+, diet sufficient in Met by dl-Met supplementation; HMTBA–, diet deficient in Met by dl-2-hydroxy-4-methylthiobutanoic acid (HMTBA) supplementation; HMTBA+, diet sufficient in Met by dl-2-hydroxy-4-methylthiobutanoic acid (HMTBA) supplementation.

^2^Equivalent total Met corresponding to the sum of Met and HMTBA transformed to Met with 84% efficiency (in a molecular equivalence basis).

^3^Supplied per kilogram of feed: Fe, 58.2 mg; Cu, 20.05 mg; Mn, 80.85 mg; Zn, 90.1 mg; I, 2 mg; Se, 0.2 mg; vitamin A (retinyl acetate), 15 000 IU; cholecalciferol, 5,000 IU; vitamin E (DL-a-tocopheryl acetate), 100 IU; vitamin K3 (menadione), 5 mg; thiamine, 5 mg; riboflavine, 8 mg; pantothenic acid, 25 mg; niacin, 100 mg; pyridoxine, 7 mg; folic acid, 3 mg; biotin, 0.3 mg; vitamin B12, 0.02 mg and choline, 550 mg.

^4^Ajusted for 87.3% DM.

^5^Total sulphur AA corresponding to the sum of the equivalent total Met and the analysed Cys.

^6^AMEn and digestible values were estimated from Sauvant et al. (2004). [Sauvant D., Perez J.M., Tran G., Tables of Composition and Nutritional Value of Feed Materials. Pigs, Poultry, Cattle, Sheep, Goats, Rabbits, Horses, Fish INRA Editions and AFZ Paris, France 2004]

^7^Estimated by the sum of the calculated Met plus the Met from HMTBA applying the 84% transformation efficiency of the HMTBA into Met.

**Supplemental Table 2** Primers used for the RT-PCR analyses

| Function | Symbol | Name | Primers | Accession number | |
| --- | --- | --- | --- | --- | --- |
| Proteolysis | *FBXO32* | F-box only protein 32 also known as atrogin-1 | 5' -GACGCGCTTTCTCGATGAG- 3' | | NM_001389309.1 |
|  |  |  | 5' -CCTTGTTATTCAGTAGGTCTTTTTTCCT- 3' | |  |
|  | *TRIM63* | RING-type E3 ubiquitin transferase (or MuRF1) | 5' -TGTCTATGGGCTGCAGAGGAA- 3' | | NC_052554.1 |
|  |  |  | 5' -GGTGCTCCCCCTTCTTGAGT- 3' | |  |
|  | *UBB* | Ubiquitin | 5' -CGCACTCTGTCCGACTACAA- 3’ | | XM415105 |
|  |  |  | 5' -GCCTTCACGTTCTCAATGGT- 3’ | |  |
|  | *PSMA1* | Proteasome subunit alpha type-1 | 5' -AACACACGCTGTTCTGGTTG- 3' | | NM_205020.1 / AF027978 |
|  |  |  | 5' -CTGCGTTGGTATCTGGGTTT- 3' | |  |
|  | *CAPN2* | Calpain 2 | 5' -ACATCATCGTGCCCTCTACC- 3' | | NM_205080.1 / D38026.1 |
|  |  |  | 5' -GAGATCTCTGCATCGCTTCC- 3' | |  |
|  | *CTSB* | Cathepsin B | 5' -CAAGCTCAACACCACTGGAA- 3' | | NM_205371.2 |
|  |  |  | 5' -TCAAAGGTATCCGGCAAATC- 3' | |  |
| Autophagy | *ATG12* | Autophagy-related 12 | 5' -AGCGGAGCGTCGTTGG- 3' | | NC_052572.1 |
|  |  |  | 5' -TGGTCTTCATAATGGGGGTGTC- 3' | |  |
|  | *ATG4B* | Autophagy-related 4B | 5' -GCAGGCATTTGGGAAGAG- 3' | | NM_213573.1 |
|  |  |  | 5' -CTGGGCTATCTGGTGAATGG- 3' | |  |
|  | *UVRAG* | UV radiation resistance-associated gene protein | 5' -GGGCTCCAGGTCTACAATCA- 3' | | NM_001030839.1 |
|  |  |  | 5' -AGGAAGGGTTTGCCGTAGAT- 3' | |  |
|  | *SQSTM1* | Sequestosome 1 | 5' -AAAGGCATCCACAAGGAGCA- 3' | | XM_001233248.5 |
|  |  |  | 5' -CCTGTTCCGAGTTCTGGCAT- 3' | |  |
|  | *BNIP3* | Bcl-2/adenovirus E1B 19 kDa interacting protein 3 | 5' -CTCATTGTGACAGCCCTCCT- 3' | | XM_421829.4 |
|  |  |  | 5' -GTCTGGGGTGTTTGAAAAGGAA- 3' | |  |
|  | *MUL1* | Mitochondrial ubiquitin ligase activator of NF-κB | 5' - GAACCGAACAACCCACCTCTG- 3' | | XM_424579.4 |
|  |  |  | 5' - TTCATCACCCTCACTGCCAC- 3' | |  |
